# Reproduction and Productivity in Dairy Cattle after Abortions Both Related and Unrelated to *Coxiella burnetii*

**DOI:** 10.3390/ani13223561

**Published:** 2023-11-18

**Authors:** Guna Ringa-Ošleja, Vita Antāne, Ivars Lūsis, Lelde Grantiņa-Ieviņa, Žanete Šteingolde, Artjoms Mališevs, Aivars Bērziņš

**Affiliations:** 1 Faculty of Veterinary Medicine, Latvia University of Life Sciences and Technologies, LV-3004 Jelgava, Latvia; vita.antane@lbtu.lv (V.A.); ivars.lusis@lbtu.lv (I.L.); aivars.berzins@bior.lv (A.B.); 2 Institute of Food Safety, Animal Health and Environment BIOR, LV-1076 Riga, Latvia; lelde.grantina-ievina@bior.lv (L.G.-I.); zanete.steingolde@bior.lv (Ž.Š.); artjoms.malisevs@bior.lv (A.M.)

**Keywords:** dairy cattle, *C. burnetii*, abortion, reproduction, productivity

## Abstract

**Simple Summary:**

The bacterium *C. burnetii*, causing Q fever in humans and animals, is widespread worldwide, but the true extent of *C. burnetii* spread is not always known. Q fever in ruminants is mainly associated with abortions and reproductive disorders. However, there is a lack of research on what happens to animals after abortion and whether reproduction and productivity in animals with *C. burnetii*-related abortions differs from those with abortions that are unrelated to *C. burnetii*. In our study, we compared data on abortions, outcome of the animals after abortions both related and unrelated to *Coxiella burnetii*, and the reproduction and productivity data for animals which had aborted to the herd average parameters. We found that *C. burnetii*-related abortions are more often observed in older cows, but there were no significant differences between groups for the other compared indicators. We found that abortions, both related and unrelated to *C. burnetii* are economically unprofitable due to a high culling rate and due to the reproductive problems caused by abortions resulting in the absence of new pregnancies. When comparing the productivity data of animals that had aborted to the herd average, we found it to be lower if the animal had started a new lactation after an abortion.

**Abstract:**

*C. burnetii* is a widespread pathogen, causing abortions and reproductive disorders in ruminants. The study aimed to evaluate animal reproductive capacity and productivity after abortion, related and unrelated to *C. burnetii*. We compared data about the abortion time, the outcome of the animals after an abortion, further reproduction, and productivity for *C. burnetii*-positive (*n* = 148) and *C. burnetii*-negative (*n* = 149) aborted dairy cows and heifers. *C. burnetii*-positive animals had a positive serological response or presence of *C. burnetii* DNA at the time of abortion. *C. burnetii*-positive animals had a significantly higher number of lactations at the time of abortion. However, in the other indicators, we observed no significant differences between the groups. Comparing indicators of all the aborted animals, we found that if animals started a new lactation after abortion, they had a significantly lower milk yield, lower fat, protein, and somatic cell counts (SCCs) in milk during the standard lactation for both primiparous and multiparous cows compared to herd averages in each group. Lower SCCs can be due to animals with a high SCC being culled earlier. We found an economic disadvantage to aborting, not only because of the loss of offspring, but also because of the high culling rate and lower productivity in both primiparous and multiparous cows.

## 1. Introduction

*Coxiella burnetii*—a causative agent of Q (query) fever—is widely spread almost worldwide [1], affecting small ruminants, cattle, buffalo, camels, and wild ruminants [2,3,4,5]. 

*Coxiella burnetii*, the causative agent of Coxiellosis and Q fever, is an obligate intracellular pathogen. Animals and humans are infected with *C. burnetii* mainly through the respiratory tract; the target cells of *C. burnetii* are monocytes and macrophages [3,4,6]. After entering, *C. burnetii* multiplies in large numbers in the regional lymph nodes. Bacteriemia lasting 5–7 days follows, after which the pathogen is localized in the mammary gland and in the placenta and trophoblast of pregnant animals [7]. In case of *C. burnetii* infection in pregnant animals, the fetus can have different outcomes—abortion [8], premature delivery, stillbirth, weak offspring (APSW complex), or clinically healthy offspring with or without congenital *C. burnetii* infection [6]. There are several studies on the role of *C. burnetii* in the incidence of abortion in dairy herds [9,10,11,12,13], in which *C. burnetii* (PCR) was detected as the cause of abortion in 1.67–11.6% of cases. Studies by other authors [14,15,16] have also revealed a higher risk of abortion in seropositive animals. *C. burnetii* infection (Q fever) in farm animals is usually described as subclinical [3,17,18]. In the case of experimental infections, a self-limiting fever was observed in the acute phase of Q fever (24–48 h after *C. burnetii* inoculation) in heifers [19]. In the context of dairy farms, it is frequently observed that farm personnel often fail to recognize or make the necessary connection between the presence of fever as a clinical symptom in animals and their potential spontaneous Q fever infection [6]. This oversight results in the disease progressing to a chronic transitional phase. Postpartum metritis has also been confirmed in studies of Q fever infection, with a higher incidence of metritis in seropositive animals [20,21], questioned [11] and recognized as a relevant and necessary area for further research on a larger scale [12]. The impact of the immune response or presence of *C. burnetii* on the incidence of postpartum metritis in seronegative heifers three weeks before calving has also been described [13]. In a more recent study, the role of *C. burnetii* in clinical and subclinical endometritis and subsequent poor fertility or infertility in cows has been strongly confirmed. By identifying the *C. burnetii* antigen as the sole pathogen in endometrial macrophages of infertile cows [14], the association of the presence of *C. burnetii* with endometritis, uterine vasculitis, and fibrosis, resulting in reduced fertility or, in more severe cases, infertile animals, has also been described. In order to elucidate the direct link between Q fever infection and embryonic death within the cattle population, recent investigations have focused on contrasting the antigen phases of the specific immune response in *C. burnetii*-positive cows. This research revealed that embryonic death occurred in 18% of seropositive cows during two critical periods of pregnancy, namely 29–35 and 60–70 days [15]. Furthermore, the literature discusses Q fever-associated placental retention in cows [22], as well as placentitis induced by *C. burnetii* in small ruminants [16].

Because in the case of Q fever infection none of the clinical signs are pathognomonic [23], the diagnosis of Q fever is confirmed via laboratory tests detecting the serological response (ELISA) or presence of DNA (real-time PCR) [24]. As in Latvia there are currently no vaccines that are registered and used against *C. burnetii* in animals, a positive serological response is attributed to an infection.

Despite the above, there are still few studies on the relationship between *C. burnetii* infection and reproductive disorders, including the post-abortion period. These deficiencies may lead to overestimating the significance of *C. burnetii* shedding or the presence of antibodies linked to reproductive disorders [6]. Therefore, when evaluating an infection often present in healthy animals, we must follow a research methodology that includes an appropriate control group [6], such as the one we followed in this study. Our study aims to investigate animal reproduction and productivity rates after an abortion, both related and unrelated to *C. burnetii*.

## 2. Materials and Methods

### 2.1. Dairy Herds Selected for the Study

For the study, five Holstein breed dairy herds (A, B, C, D, E) in Latvia were selected from those tested for a *C. burnetii* serological response (ELISA) in aborted dairy cows and pregnant heifers or DNA in abortion products (PCR) from 2016 to 2019 and having both *C. burnetii* positive and negative results. The *C. burnetii* serological response and the DNA in the abortion products were examined under The National Surveillance Program of Infectious Diseases in Food Safety, Animal Health, and Environment Institute BIOR.

Dairy herds included in the study were housed in free-stall barns and fed with total mixed ration. The average numbers of heifers from 2016 to 2019 were 282, 58, 184, 290, and 363, the average numbers of dairy cows were 658, 154, 387, 534, and 728, and the average milk yields were 11427.0, 8709.6, 9572.4, 8096.8, and 10,430.8 kg in A, B, C, D, and E herds, respectively. 

Over the course of four years, from 2016 to 2019, researchers examined sera from 361 individuals and analyzed abortion products, including fetuses, fetal organs, and placental tissues, obtained from 103 cows and heifers that had experienced abortions. In total, 464 animals were tested. The number of blood samples examined from herds A, B, C, D, and E were 201, 30, 33, 30, and 67, respectively. The number of abortion products examined from herds A, B, C, D, and E were 55, 23, 11, 14, and 1, respectively. 

### 2.2. Laboratory Analysis of the Sera and Abortion Material

Sera were tested using ID Screen Q Fever Indirect Multi-Species ELISA (ID Vet, Grabels, France). Abortion products were tested using the ADIAVET COX REAL-TIME test to detect *Coxiella burnetii* via real-time enzymatic DNA amplification (PCR test) (Bio-X Diagnostics, Rochefort, Belgium). Tests were carried out following the manufacturer’s instructions.

From 361 sera samples tested, in total, we found 139 (38.50%) *C. burnetii* seropositive animals. At the herd level, we found 84 (32.81%), 22 (41.51%), 15 (34.09%), 9 (20.46%), and 12 (17.65%) *C. burnetii* positive animals from A, B, C, D, and E herds, respectively. 

From the 103 abortion products tested, in total we found 36 (34.95%) *C. burnetii* DNA-positive animals. At the herd level, we found 28 (50.91%), 5 (21.74%), 0 (0%), 3 (21.43%), and 0 (0%) *C. burnetii* positive animals from A, B, C, D, and E herds, respectively. 

### 2.3. Animals Included in the Study and Parameters Compared

In this study, we used individual reproduction and productivity data from all PCR-positive (*n* = 36) and PCR-negative (*n* = 67) animals and ELISA-positive (*n* = 112) and ELISA-negative (*n* = 82) animals, selected using a systematic random sampling method. The selection of animals for the study was based solely on the results of either ELISA or PCR testing. 

Thus, in total, we had a *C. burnetii*-positive group, having a positive serological response or presence of *C. burnetii* DNA at the time of abortion (*n* = 148), including 29 pregnant heifers and 119 milking cows (from first to seventh lactation), and a *C. burnetii*-negative group (*n* = 149), including 38 pregnant heifers and 111 milking cows (from the first to eighth lactation). Since no mandatory culling policy is provided for animals in cases of positive testing for *C. burnetii*, either ELISA and PCR, three possible outcomes after an abortion were detected and described: culling within 14 days after abortion, the same lactation with or without a following successful artificial insemination (AI), or new lactation both in the heifers becoming primiparous cows and in milking cows having late-term abortions during a dry period. 

Individual data pertaining to abortion, including the lactation stage and pregnancy duration at the time of abortion, as well as information on the subsequent fate of the animals (such as culling, remaining in the same lactation, or entering a new lactation), reproductive performance metrics (such as the number of artificial AI, pregnancy rate after the first AI, occurrences of prolonged estrous cycles exceeding 23 or 48 days, days open, days until the first AI, pregnancy rate up to 150 days postpartum, and the status of offspring), and productivity metrics during the standard lactation (including milk yield, fat content, protein content, and SCC) following an abortion, were collected and analyzed in this study. SCC data were converted into log (2) units, as described by Shook, 1993 [25], and Shook, 1993 [26]. 

Those reproductive parameters not summarized at the herd level (pregnancy rate after the first AI, prolonged cycles above 23 or 48 days, days until the first AI, pregnancy rate until 150 days after delivery, and status of offspring) were compared only between *C. burnetii*-positive and -negative groups. All the numbers of compared parameters can be found in the supplemental table (Table S1).

### 2.4. Statistical Analysis

The descriptive statistics (median) were calculated using Jamovi (Version 2.4, 2023). The median number of lactations, pregnancy duration at the time of abortion, and reproductive parameters were compared using the Mann–Whitney U test (Jamovi, Version 2.4, 2023).

To compare frequencies of age group (heifer, 1st lactation, 2nd, and higher lactation), of pregnancy trimester (1st, 2nd, and 3rd trimester) at the time of abortion, and the outcome of an animal after an abortion (culled, the same lactation, new lactation), we used the χ^2^ test (Jamovi, Version 2.4, 2023). 

To assess the reproductive and productivity parameters between *C. burnetii*-related and unrelated abortion groups, we employed a two-sample *t*-test, assuming within group independence of observations. To assess the reproductive and productivity parameters of each aborting animal in comparison to the herd’s average data for the same year, we employed a one-sample *t*-test. In addition, we examined the *C. burnetii*-positive status, the commencement of a new lactation, and the influence of individual herd-specific characteristics on all productivity parameters, as well as the individual deviations from each herd’s average parameters, including milk yield (kg), fat (kg), protein (kg), and SCC {log (2)} units during the standard lactation period of 305 days. This comprehensive analysis was conducted using multivariate analysis of variance MANOVA (StataCorp LP, 4905 Lakeway Drive, College Station, TX 77845, USA, version Stata BE 18.0 for Windows). 

Results with *p* < 0.05 were considered significant.

## 3. Results

### 3.1. Time of Abortion

**Figure 1:**
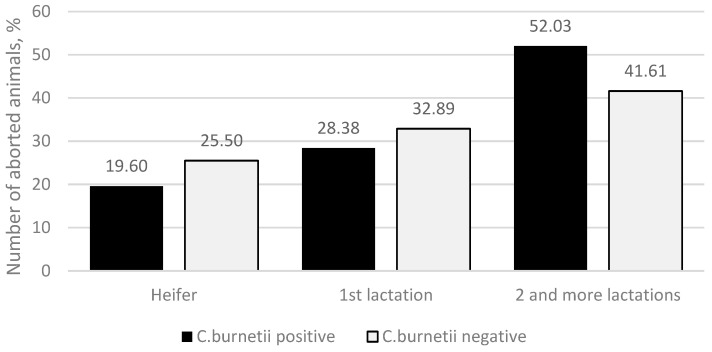
Number of aborted animals by number of lactations (heifers, 1st lactation, 2 and higher lactations). The *y*-axis indicates the percentage of *C. burnetii*-positive (black column) and *C. burnetii*-negative (light-gray column) aborted animals.

The median number of lactations in which abortions were detected in the group of *C. burnetii*-positive animals was higher (*p* = 0.048), showing 2 lactations, and in the group of *C. burnetii*-negative animals, 1 lactation, respectively. A more detailed study of the data on the number of lactations in which abortion occurs showed that in the *C. burnetii*-positive group, 29 (19.60%) abortions were found in heifers, 42 (28.38%) in the primiparous cows, and 77 (52.03%) in the second and higher lactation cows. In the *C. burnetii*-negative group, 38 (25.50%) abortions were found in heifers, 49 (32.89%) in the primiparous cows, and 62 (41.61%) in the cows with a second or higher lactation (Figure 1), and no significant differences were observed (*p* = 0.186).

**Figure 2:**
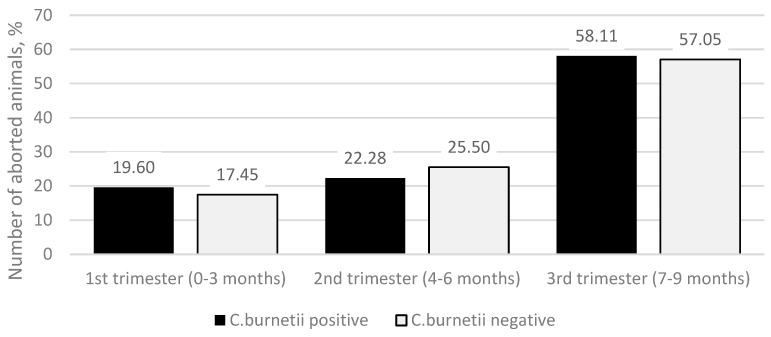
Duration of pregnancy (months) at the time of abortion (1st trimester (0–3 months), 2nd trimester (4–6 months), and 3rd trimester (7–9 months). The *y*-axis indicates the percentage of *C. burnetii*-positive (black column) and *C. burnetii*-negative (light-gray column) aborted animals.

The median value of the duration of pregnancy in months at the time of abortion in both *C. burnetii*-positive and -negative groups was 7 months. A more detailed study of the data on the month of pregnancy in which abortion occurs (Figure 2) showed that in the *C. burnetii*-positive group, there were 29 (19.60%) abortions in the first trimester (0–3 months of pregnancy), 33 (22.28%) abortions in the second trimester (4–6 months of pregnancy), and 86 (58.11%) abortions in the third trimester (7–9 months of pregnancy). In the *C. burnetii*-negative group, there were 26 (17.45%) abortions in the first trimester, 38 (25.50%) in the second trimester, and 85 (57.05%) third-trimester abortions. The differences between the groups were not statistically significant (*p* = 0.772).

### 3.2. Outcome of the Animal after Abortion

**Figure 3:**
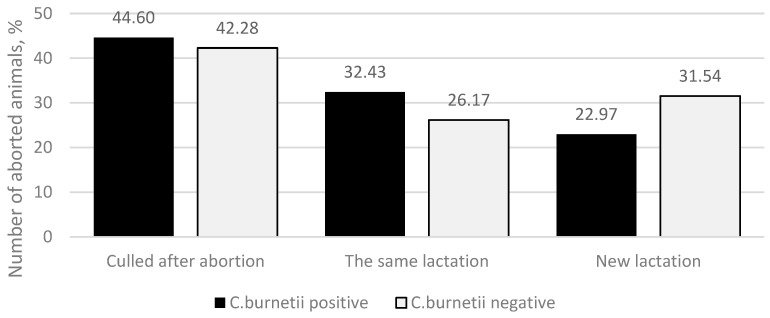
Possible outcomes in animals after abortion (culled after abortion, the same lactation, new lactation). The *y*-axis indicates the percentage of *C. burnetii*-positive (black column) and *C. burnetii*-negative (light-gray column) aborted animals.

In the study, when summarizing the outcomes for animals after abortion, three were found: the animal was culled in the following 14 days after the abortion, the same lactation continued, or a new lactation was initiated (Figure 3). In the *C. burnetii*-positive group, 66 (44.60%) animals were culled in the following 14 days after abortion, 48 (32.43%) animals continued the same lactation, and 34 (22.97%) animals initiated a new lactation. In the *C. burnetii*-negative group, 63 (42.28%) animals were culled in the following 14 days after an abortion, 39 (26.17%) animals continued the same lactation, and 47 (31.54%) animals initiated a new lactation. The differences between the groups in each outcome were not statistically significant (*p* = 0.214).

**Figure 4:**
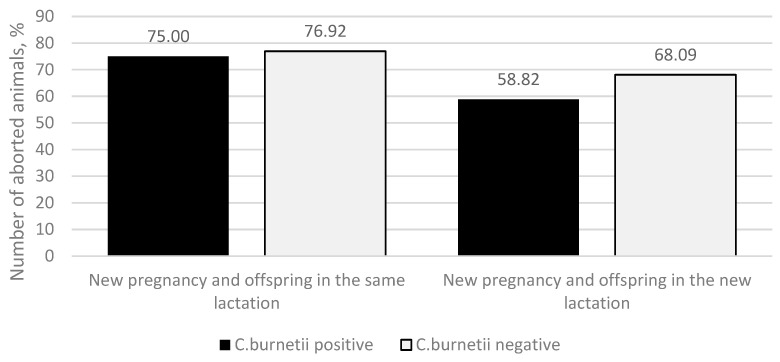
The onset of a new pregnancy during the same or new lactation (new pregnancy and offspring in the same lactation, new pregnancy and offspring in the new lactation). The *y*-axis indicates the percentage of *C. burnetii*-positive (black column) and *C. burnetii*-negative (light-gray column) aborted animals.

By continuing to study the data on animals that continued the same lactation or initiated a new one, we found that in both cases, two possibilities followed—after the abortion, a new pregnancy occurred, followed by calving (Figure 4), or a new pregnancy did not occur, and the animals were culled as non-pregnant.

If the animal had the same lactation after the abortion, *C. burnetii*-positive animals had fewer new pregnancies compared to the *C. burnetii*-negative ones (36 (75.00%) and 30 (76.92%) animals, respectively). That was observed along with the higher culling rate of non-pregnant animals (12 (25.00%) and 9 (23.08%) animals, respectively). The same we observed if a new lactation started after the abortion—*C. burnetii* positive animals had fewer new pregnancies after the abortion compared to the *C. burnetii*-negative ones (20 (58.82%) cows and 32 (68.09%) cows, respectively). Those non-pregnant in the new lactation, 14 (41.18%) *C. burnetii*-positive animals and 15 (31.91%) *C. burnetii*-negative animals, were culled. The differences between groups were not statistically significant (*p* = 0.298). In summary, from the initially 148 *C. burnetii*-positive animals, there were 56 new offspring (of which 51 were alive), and from *C. burnetii*-negative animals, 62 new offspring (of which 53 were alive) were produced by pregnancy after an abortion. This difference was found to be statistically insignificant (*p* = 0.814).

### 3.3. Reproduction Rates Comparing C. burnetii-Positive and C. burnetii-Negative Groups

We found no statistically significant differences comparing reproduction rates for animals having the same lactation (Table 1) or initiating a new lactation (Table 2).

### 3.4. Reproduction and Productivity Rates Comparing All Aborted Animals versus Herd Averages

Based on the finding of no significant differences in reproduction rates between the *C. burnetii*-positive and *C. burnetii*-negative groups, we compared the individual reproduction and productivity rates of each aborted animal against the average in the herd of this animal for the corresponding year (Table 3 and Table 4).

For cows having the same lactation after abortion, open days were 143.30 ± 11.70 days more than the herd average of the relevant year. This difference was significant (*p* < 0.05). The number of artificial inseminations per pregnancy in aborted animals did not differ from the herd averages. The milk yield, fat, and protein content in a standard lactation (305 days) of the aborted animals were at the average level of the herd. The SCC in milk in a standard lactation was 0.83 ± 0.17 log (2) units or 1.74 times lower (*p* < 0.05) than in cows of a similar age (lactation) in the same herd.

In aborted animals that started a new lactation after the abortion, no differences were found in the open days and in the AI rate per pregnancy from the average herd values. When evaluating the milk yield in a new lactation, all aborted cows, regardless of *C. burnetii* status, can be predicted to have an average of 1523.20 ± 292.80 kg lower milk yield, an average of 54.90 ± 12.00 kg lower fat content, and an average of 43.30 ± 9.40 kg lower protein content than in a standard lactation, and the difference was significant (*p* < 0.05). In the new lactation, SCC was 1.30 ± 0.17 log (2) units or 2.4 times lower (*p* < 0.05) than in cows of a similar age (lactation) in the same herd. The milk yield of the aborted heifers (*n* = 35) was significantly lower (−1730.65 ± 412.87 kg) than the average milk yield in primiparous cows of their herd. A decrease in the milk yield by ~1300 or ~1400 kg was also observed in the cows of the second, third, and higher lactations, compared to the average milk yield of the other cows of the given herd. In the data collection, we found that a new lactation can also be shortened (on average 292.74 ± 3.53 days). However, this did not explain the significant reduction in the standard lactation milk yield by ~1500 kg. Abortions in heifers were observed on average 2.12 ± 0.27 months before the herd’s average age of the first calving. As mentioned, some of the aborted heifers were culled, but those that started a new lactation after the abortion (*n* = 35) had a similar delivery time, 2.31 ± 0.28 months earlier than the herd’s average.

In summary, abortions related to *C. burnetii* were observed in older cows (with more lactations), starting from two or more lactations, but in at least 19.60% of cases, it also occurred in heifers. It was found that more than half of the investigated abortion cases were detected in the last trimester of pregnancy. However, no significant differences were observed between the *C. burnetii*-positive and *C. burnetii*-negative groups. When evaluating the impact of abortions on the future use of the animal, we found that 44.60% of *C. burnetii*-positive aborted animals were culled within 14 days after the abortion, 32.43% continued the same lactation, and 22.97% of the animals (including pregnant heifers) started a new lactation. After the abortion, not all the animals became pregnant again, even after several AIs; therefore, they were culled as non-pregnant. Thus, out of 148 *C. burnetii*-positive animals included in this study, a new pregnancy after an abortion occurred and ended with the calving of 56 cows (51 live offspring), while in the 149 *C. burnetii*-negative animals, a new pregnancy after abortion occurred and ended with the calving of 62 cows (53 live offspring). No significant differences were found in the evaluated reproduction rates after abortion between *C. burnetii*-positive and *C. burnetii*-negative groups. However, when we compared the further reproduction and productivity rates of all 297 aborted animals versus the herd-averaged rates of the relevant year, we found more open days in the aborted animals which continued the same lactation. In cows that started a new lactation following an abortion, there was a lower milk yield, milk fat and protein content, and SCC in a standard lactation.

## 4. Discussion

The analysis of the average number of lactations when abortions occurred revealed a statistically significant difference between the *C. burnetii*-positive and *C. burnetii*-negative groups. In the *C. burnetii*-positive group, the median value was 2 lactations, whereas in the *C. burnetii*-negative group, the median value was 1 lactation (*p* = 0.048). Notably, the highest incidence of *C. burnetii* in abortion cases was observed among cows with two or more lactations. Paul, 2014, [27] also found a higher incidence of *C. burnetii* in older animals because the number of lactations is an indirect indicator of the age of the animals. Also, a significant increase in the proportion of *C. burnetii* immune response and positive animals in the first lactation in initially seronegative heifers has been described [28]. Thus, this explains our result that heifers and primiparous cows become infected in common parturition areas from older cows spreading pathogens.

The median value of duration of pregnancy in months at the time of abortion was 7 months in both *C. burnetii*-positive and -negative groups. When evaluating the distribution of the incidence of abortion by trimester of pregnancy, we found that in both groups, it was the highest in the last trimester of pregnancy (7–9 months), which can be explained by the fact that the fetuses of late abortions are examined more often simply because early abortions (up to a 4-month-long pregnancy) are not seen or reported on farms [8].

When evaluating the outcome of animals after abortion, we found that 66 (44.60%) *C. burnetii*-positive and 63 (42.28%) *C. burnetii*-negative animals were culled in the following 14 days after having an abortion. Our findings agree with those of Ansari-Lari et al., 2012 [29], in which 28% of animals were culled within the first 100 days after calving, and the average number of days after calving to culling was 240. In our study, we observed the culling of inseminated but non-pregnant animals within the following 240 days. It is known that each case of abortion causes economic losses due to lost offspring, an extended inter-calving interval, and an earlier removal of productive animals from the herd, and it is estimated that the abortion of a dairy cow causes a loss of approximately USD 1415 [30]. In our study, abortion was a significant reason for the culling of both cows and heifers.

In both groups, animals not culled in the following 14 days after abortion received artificial insemination. However, they were culled if pregnancy did not occur after multiple artificial inseminations (the maximum detected number of AIs was 7). This finding pointed to post-abortion infertility as one of the predominant reasons for culling and agreed with other studies finding infertility [28,29,30,31], mastitis, high SCC in milk [32], low productivity, and leg problems [33] as the most common reasons for culling.

Comparing the AI rate per new pregnancy in groups, we did not find significant differences (1.67 ± 0.21 AI events for *C. burnetii*-positive and 1.60 ± 0.16 AI events for *C. burnetii*-negatives, respectively). When evaluated among all aborted animals having the same lactation after abortion, the AI rate was 1.63 ± 0.14 times. Among animals that started a new lactation after abortion, the AI rate was 2.20 ± 0.23 times. The differences from the herd averages (1.86 ± 0.03 and 1.99 ± 0.04, respectively) were not statistically significant (*p* > 0.05). The AI rate is considered good at 1.6 times, 1.6–1.8 times is average, around two times is acceptable, while three or more AI times per pregnancy is considered a bad indicator [34]. It is known that the number of inseminations per pregnancy and the number of days from calving to the first AI increases with the age of the animals [35]. Therefore, in our study, the AI rate after an abortion was found to be acceptable.

When comparing the onset of a new pregnancy, it was lower in the *C. burnetii*-positive group in both the same lactation (36 (75.00%) animals) and when starting a new lactation (20 (58.82%) animals). However, differences in the *C. burnetii*-negative group (30 (76.92%) and 32 (68.09%) animals, respectively) were not statistically significant (*p* = 0.814).

Our study assessed the intervals between repeated AIs in animals having new pregnancies after abortion (both same and new lactations). If they exceeded the length of a regular cycle (above 23 days or above 48 days) [36], they were defined as prolonged cycles, indicating embryonic death [37]. Our study observed prolonged cycles above 23 days in 16.67–20.00% of *C. burnetii*-positive and 6.67–18.75% of *C. burnetii*-negative animals. Prolonged cycles above 48 days were observed in 16.67–20.00% of *C. burnetii*-positive and 16.67–21.88% of *C. burnetii*-negative animals. No significant differences between groups were found. To explain the embryonic death directly related to Q fever infection in the herd, studies in recent years found embryo death (29–35 and 60–70 days of pregnancy) in 18% of *C. burnetii*-seropositive cows [38]. Thus, our findings are similar to those mentioned. A very high prevalence of seropositivity was found in 80.5% of cows and 94.4% of heifers with the early termination of pregnancy. A higher prevalence of *C. burnetii* phase I antigen seropositivity (50.0%) was found in cows with an abortion than in cows with a maintained pregnancy (38.5%) [15], indicating the adverse effect of *C. burnetii* on maintaining the pregnancy. In our study, we found relatively high rates of early pregnancy loss in cows after abortion, both related and unrelated to *C. burnetii*. Therefore, further studies may be necessary for more extensive evidence.

When late abortions, followed by the commencement of a new lactation, were considered as calving events, our analysis did not reveal any statistically significant differences between the groups in various parameters. Specifically, there were no significant differences in terms of time to the first AI, open days, the initiation of pregnancy after the first AI, or the initiation of a new pregnancy within 150 days (*p* > 0.05). It agrees with study [13], in which no significant differences were found in animals tested for the immune response in serum (ELISA) and the presence of *C. burnetii* in vaginal excretions and milk (PCR) in these indicators. In a pilot study [12], using the ovulation synchronization protocol and performing the first AI around 70 days after calving did not lead to worse fertilization rates after the first AI in cows with a positive *C. burnetii* immune response. However, they emphasized the need to continue the research on a larger scale. Pregnancy after the first AI is reported in 34% [39] to 50% of dairy cows [40]. In our study, if abortion initiated a new lactation, it was 50.00%—the same in both groups. It should be noted that this indicator was even higher in animals that, after the abortion, had the same lactation: 60.00% in the *C. burnetii*-positive group and 69.44% in the *C. burnetii*-negative group (the difference is statistically insignificant (*p* > 0.05)). Considering the fact of abortion, in our study, this high rate was not unequivocally evaluated as optimal or satisfactory. However, it showed that even after abortion, the insemination of animals was successful. A study finds better reproductive performance in Q fever seropositive animals compared to uninfected ones [41].

When evaluating the productivity rates, we did not observe any significant differences between the *C. burnetii*-positive and *C. burnetii*-negative groups (*p* > 0.05). When evaluating the rates of all aborted animals against the herd’s average ones of the respective years, we found that in both scenarios, having the same lactation or starting a new one, the SCC was significantly lower in aborted cows compared to the herd averages (*p* < 0.05). As Crowe, 2016, [36] describes, cows with a high SCC have a higher incidence of reduced reproductive performance. The lower SCC in our study can be explained by cows having a high SCC failing to become pregnant again and thus have been culled. When starting a new lactation, both the milk yield and the content of fat and protein in the milk in a standard lactation of aborted animals was significantly lower (*p* < 0.05). Evaluating in more detail, we found that the enormous milk yield lag (1523.20 ± 292.80 kg) from the herd’s average rate was observed in the primiparous cows (pregnant heifers whose first lactation started with an abortion), which could be explained by the fact that abortions in these heifers (*n* = 35) were observed on average 2.31 ± 0.28 months before the herds’ average first calving age (24.17 ± 0.18), which indicates the physiological unpreparedness of the animals to start lactation. Heifers with their first calving as early as 22–23 months of age (after full gestation) are known to show the best milk yield and survival rates during the first five years [42]. However, in our study, the heifers began lactation earlier due to abortion, thus showing a lower productivity in the first standard lactation. Interestingly, the adverse impact of commencing lactation with an abortion on future productivity was not limited to first-lactation cows. It extended to other cows that had experienced abortions, resulting in a notable reduction of 1300–1400 kg in milk yields during the standard lactation period compared to the herd’s overall average. Keshavarzi et al., 2020, [43] also describes about 7–19% lower milk production after abortion. This decline in milk production underscores the unpreparedness of these animals for initiating a new lactation. Mainly, the dry period is observed to be around 60 days. However, it has been found that a shortened one (40 days), if it is specially managed, has a positive effect on the further use of animals, and the milk yield is not affected [42,44]. On the other hand, if the dry periods are shortened due to premature delivery (including abortions and twin pregnancies), the future milk yield is predicted to be lower [45]. Earlier initiation of lactation in such cases is also associated with the risk of drug residues in milk if intramammary antibacterial drugs were administered to the animal before the dry period.

## 5. Conclusions

The incidence of *C. burnetii*-related abortions was notably more frequent among older cows, exhibiting a median of 2 lactations. In contrast, *C. burnetii* unrelated abortions showed a median of only one lactation, and this disparity was statistically significant (*p* = 0.043). Consequently, older cows exhibit an elevated propensity to act as potential *C. burnetii* carriers, thereby posing a greater risk of infecting heifers in shared calving areas.

Despite the higher culling rate observed in cows that experienced abortions related to *C. burnetii* (totaling 92 animals), the difference did not reach statistical significance when compared to cows that had abortions unrelated to *C. burnetii* (totaling 87 animals) (*p* > 0.05). Furthermore, various reproductive parameters, including pregnancy following the first AI, AI rate per pregnancy, instances of prolonged cycles (both exceeding 23 and 48 days), open days, days elapsed after abortion until the first AI, and the initiation of a new pregnancy within 150 days post-abortion, displayed no significant differences between the *C. burnetii*-related and unrelated groups (*p* > 0.05). Thus, we can conclude that abortions related to *C. burnetii* had the same impact on the future reproductive performance of animals as abortions linked to other causative factors.

In the comparison of reproductive and productivity data for all animals that experienced abortions to the herd’s average parameters, a notable increase in the duration of open days was identified in the aborting animals, measuring at 143.30 ± 11.70 days, compared to the herd’s average (*p* < 0.05). In cases where animals initiated a new lactation following an abortion, our study revealed significantly reduced levels of milk yield, fat content, protein content, and SCC in the aborted animals when compared to the herd’s overall average (*p* < 0.05). This finding highlights the detrimental impact of an abortion on the premature commencement of lactation and its subsequent consequences on productivity, affecting both groups related and unrelated to *C. burnetii.*

## Figures and Tables

**Table 1:** The reproduction rates in animals having the same lactation after abortion.

Reproduction Rate	*C. burnetii*-Positive Animals (*n* = 36)	*C. burnetii*-Negative Animals (*n* = 30)
Pregnancy after the 1st AI, *n* (%)	25 (69.44)	18 (60.00)
AI rate per pregnancy, mean ± SE	1.67 ± 0.21	1.60 ± 0.16
Prolonged cycles above 23 days, *n* (%)	6 (16.67)	2 (6.67)
Prolonged cycles above 48 days, *n* (%)	6 (16.67)	5 (16.67)
Days open, mean ± SE	265.00 ± 16.90	276.00 ± 17.50

**Table 2:** The reproduction rates in animals initiating a new lactation after abortion.

Reproduction Rate	*C. burnetii*-Positive Animals (*n* = 20)	*C. burnetii*-Negative Animals (*n* = 32)
Pregnancy after the 1st AI, *n* (%)	10 (50.00)	16 (50.00)
AI rate per pregnancy, times ± SE	2.10 ± 0.28	2.25 ± 0.34
Prolonged cycles above 23 days, *n* (%)	4 (20.00)	6 (18.75)
Prolonged cycles above 48 days, *n* (%)	4 (20.00)	7 (21.88)
Days open, mean ± SE	115.00 ± 13.10	122.00 ± 14.50
Days after abortion until the 1st AI, mean ± SE	76.30 ± 5.72	73.10 ± 4.89
Onset of new pregnancy until 150 days after abortion, *n* (%)	16 (80.00)	25 (78.13)

**Table 3:** The reproduction and productivity rates of aborted animals having the same lactation after abortion and the average rates in the herd.

Reproduction/ Productivity Rate	Aborted Animals (*n* = 63), Mean ± SE	Average Rate in the Herd, Mean ± SE
Open days	269.92 ± 12.08 *	126.57 ± 1.57
AI rate per pregnancy	1.63 ± 0.14	1.83 ± 0.03
Milk yield in standard lactation (305 days), kg	10,557.25 ± 323.95	10,357.70 ± 129.89
Milk fat in standard lactation (305 days), kg	420.26 ± 12.31	410.33 ± 4.49
Milk protein in standard lactation (305 days), kg	352.23 ± 9.78	344.86 ± 3.30
SCC in standard lactation (305 days), 1000 cells/mL	101.10 ± 19.65	104.06 ± 5.59
SCC, log (2) units	2.14 ± 0.18 *	2.97 ± 0.06

* *p* < 0.05.

**Table 4:** The reproduction and productivity rates of aborted animals starting new lactation after abortion and the average rates in the herd.

Reproduction/ Productivity Rate	Aborted Animals (*n* = 54), Mean ± SE	Average Rate in the Herd, Mean ± SE
Open days	118.92 ± 10.19	129.02 ± 1.61
AI rate per pregnancy	2.20 ± 0.23	1.99 ± 0.04
Milk yield in standard lactation (305 days), kg	8645.44 ± 377.00 *	10,189.15 ± 150.20
Milk fat in standard lactation (305 days), kg	346.95 ± 15.78 *	403.25 ± 6.34
Milk protein in standard lactation (305 days), kg	297.25 ± 12.12 *	341.51 ± 3.94
SCC in standard lactation (305 days), 1000 cells/mL	76.08 ± 16.59 *	111.26 ± 6.65
SCC, log (2) units	1.75 ± 0.19 *	3.05 ± 0.07

* *p* < 0.05.

## Data Availability

The datasets generated and/or analyzed during the current study are not publicly available but are available from the corresponding author on reasonable request.

## Supplementary Materials

The following supporting information can be downloaded at: https://www.mdpi.com/article/10.3390/ani13223561/s1.
